# Promoting allied health profession careers to school students: a case study evaluation of a widening participation outreach intervention

**DOI:** 10.1186/s12909-026-10091-3

**Published:** 2026-08-07

**Authors:** Maria Wishart, Luke Capon, Nicola Alexander, Sally Fowler-Davis

**Affiliations:** 1 Centre for Health and Care Research, Anglia Ruskin University, Bishop Hall Lane, Chelmsford, CM1 1SQ UK; 2 East Suffolk & North Essex NHS Foundation Trust, Ipswich Hospital, Heath Road, Ipswich, IP4 5PD UK

**Keywords:** Allied Health Professions (AHPs), Widening participation, Outreach, Recruitment, Case study

## Abstract

**Background:**

Against a backdrop of chronic staff shortages, a situation that is amplified in rural and coastal regions of England, the NHS workforce plan expresses ambitions to increase the number of Allied Health Professional (AHP) training places. It also aims to enhance the scope of some AHP roles to enable them to improve the sustainability of service provision. The East Suffolk & North Essex NHS Foundation Trust (ESNEFT) has recently implemented a trial intervention programme to engage directly with school pupils and offer them experiential learning about AHP careers, in order to support local recruitment efforts and widen participation. This study aimed to explore participants’ experiences of the programme, to evaluate its relevance, coherence, effectiveness and impacts.

**Methods:**

This study is an evaluation of the intervention programme. We adopted a case study approach using both qualitative and quantitative data sources to explore how participants experienced and interpreted the intervention programme. We generated qualitative data from in-depth interviews, and quantitative data from a participant survey to explore their experiences.

**Results:**

We found that a substantial proportion of participants had little or no prior knowledge of AHPs before joining the programme. Participants particularly appreciated the interactive nature of the programme and the exposure to clinical settings and equipment. They were also very engaged with, and impressed by, the AHPs who delivered the content. Having taken part in the programme, participants reported increased likelihood of considering an AHP career in the future.

**Conclusions:**

We conclude that by offering authentic exposure to clinical environments and practitioners, the programme engaged participants positively with AHP professions. This may in turn increase their interest in AHP careers. We point to the role of exposure to professional role models, which may foster participants’ development of possible future selves as AHPs, in these positive outcomes.

**Supplementary Information:**

The online version contains supplementary material available at https://doi.org/10.1186/s12909-026-10091-3.

## Background

### Allied health professionals

Allied Health Professionals (AHPs) in the UK include fourteen degree-level educated autonomous healthcare professions (art therapists, dietitians, drama therapists, music therapists, occupational therapists, operating department practitioners, orthoptists, osteopaths, paramedics, physiotherapists, prosthetists & orthotists, podiatrists, radiographers, speech & language therapists) [1]. With 152,000 practitioners across all healthcare settings [1] AHPs are the third-largest workforce in the National Health Service (NHS) [2]. Their primary aim is the improvement of health and wellbeing to maximise the potential for individuals to live full and active lives [3] which resonates strongly with the UK government’s ambition to shift the focus of NHS care to prevention and early intervention [4]. Chronic staffing shortfalls in the UK NHS are well documented, with recent statistics pointing to an overall vacancy rate of 6.7%, or around 100,000 vacancies, in September 2025 [5]. While there is no published data specifically on AHP vacancies, evidence does indicate a recent decline in previous achievements to increase AHP numbers in the UK [6]. AHP shortages are acutely felt, impact on service delivery and are expected to continue to increase, particularly for paramedics, occupational therapists, diagnostic radiographers, podiatrists, and speech and language therapists. This is largely due to rising service demand outpacing workforce supply [4].

In the context of these shortages, and of the government’s stated aim of moving healthcare delivery from hospital-based services to community-based care, with a greater emphasis on prevention rather than acute treatment, the NHS workforce plan [4] expresses ambitions to increase the number of AHP training places by 25% to over 18,800 by 2032. It also aims to enhance the scope of some AHP roles to enable them to improve the sustainability of service provision through advanced practice and extended clinical responsibilities. A better understanding of AHP recruitment challenges, and interventions that may address them, could contribute significantly to delivering these ambitions.

This study evaluates one such intervention. A pilot outreach programme was undertaken by the East Suffolk & North Essex NHS Foundation Trust, with the aim of engaging school students with AHP careers and attracting more locally-based applicants. The intervention programme was designed to address cultural, financial, and informational barriers that can deter young people from pursuing careers in the Allied Health Professions, while actively contributing to the development of a sustainable, locally rooted workforce pipeline. Although modest in scale, the initiative worked directly with schools engaging pupils, raising awareness of AHP career pathways within the local community and offering hands-on experiential learning about these professions. By focusing on school-based engagement, the programme sought to generate interest and aspiration among students who are more likely to remain connected to the area, thus establishing a local supply chain of future AHPs. In doing so, it aimed not only to increase access to AHP careers but also to enhance the likelihood that participants may go on to train, work, and build long-term careers within the region, supporting workforce sustainability for both the Trust and the wider NHS. This case study evaluation aims to explore participant experiences of the intervention and its impact on their career ambitions.

### Recruiting students into allied health professions

While there is a substantive body of research on the healthcare workforce, it has a strong focus on retention, attrition, and working conditions of qualified staff, with limited empirical attention on understanding why students choose to enter healthcare professions [7, 8]. Research relating to the attraction and recruitment of future healthcare professionals has been more extensively developed within nursing and medicine than within the AHP professions [9]. For example, extant research indicates that barriers including challenging working conditions and lower starting salaries may help to explain the relatively low proportion of nursing recruits accounted for by school leavers [9, 10]. Beattie et al. [11] suggest that immersive pre-recruitment experiences may improve levels of school leaver recruitment into nursing careers. However, Clipstone and Ambrosio [10] find that observing and practising clinical skills often forms part of such experiences but argue that a prior interest in nursing as a career is often a precursor to engaging with such initiatives and point to a lack of robust evidence on the long-term impact of such programmes on actual recruitment.

The very limited literature focusing specifically on AHP recruitment identifies a lack of awareness of AHP roles among school leavers and, perhaps relatedly, limited role models in individuals of a similar background as barriers to the recruitment of school students [12]. Low levels of awareness of particular AHP professions including speech and language therapy and physiotherapy, and an associated lack of understanding of the scientific nature of the role has found to be exacerbated in underrepresented groups, which may contribute to the lack of gender and ethnic diversity in the AHP workforce [13, 14]. Engaging with students at a time when they are actively starting to consider possible career paths through targeted interventions has been suggested in prior studies [15]. Making school career advisers aware of healthcare career paths beyond medicine and nursing may also have a role to play [11] and early trials of work experience in specific Allied Health Professions have been positively received by students and AHPs [16].

The sparse evidence in this area suggests that attracting school-leavers to healthcare careers requires a holistic approach combining intrinsic motivation, early experiential learning, role model engagement, and addressing financial and perception barriers. Targeted, inclusive strategies may well have a role in increasing the pool of young, committed entrants into allied health professions, and understanding how and why interventions work will be key to progressing this agenda.

### The intervention

Entitled ‘Next AHP’, the intervention programme was modelled on a previous initiative called ‘Next Medic’, a 5-year programme designed to support young people meeting widening participation criteria onto medical doctor programmes. The ‘Next Medic’ model and experiential learning from previous outreach events promoting AHP careers to young people informed the development of the ‘Next AHP’ programme. The programme targeted year 12 students, typically aged 16 to 17, currently studying level 3 science A-levels or health and social care courses, who were aspiring to a career in health and care, but yet to decide a specific profession. Recruitment prioritised students from traditionally underrepresented backgrounds. Appendix 3 shows the widening participation criteria used. The programme offered six half day interactive sessions which were run on Saturday mornings. The sessions showcased the AHP professions through case studies, access to clinical environments and face-to-face presentations from, and engagement with, AHPs themselves. It also offered access to work experience, mentoring, university application and job application support. The programme particularly aimed to raise awareness of less well known AHP professions, which can be more difficult to recruit to. The pilot presentation started in September 2024 with a cohort of 34. Sessions were generally well-attended, but attendance fell away once the students reached the end of year 12 and the beginning of year 13, which was attributed to scheduling conflicts between the Saturday morning sessions and university open days.

## Methods

### Aims

The study had three objectives:

1. To evaluate the relevance, coherence, effectiveness and impact of the 'Next AHP' programme, through an exploration of participants’ experiences.
2. To elicit participant’s views on the strengths and limitations of the programme.
3. To understand whether/how 'Next AHP' has influenced participants’ attitudes towards AHP or broader health and care-related careers.

### Study design

This study employed a convergent mixed-methods case study design to evaluate the programme [17]. Qualitative data were collected through semi-structured interviews and analysed inductively using thematic analysis supported by the qualitative data software analysis package NVivo 14 while quantitative data were collected through a survey and analysed using descriptive statistics. Qualitative data collection and analysis were undertaken first, followed by a quantitative phase. The two datasets were analysed independently before being integrated during interpretation to develop a comprehensive understanding of the relevance of the programme, its strengths and limitations and its impact on the future career intentions of the participants.

A case study evaluation uses in‑depth case study methods to examine how, why, and in what contexts a programme, policy, service, or intervention works (or does not work). The case for this study is a pilot outreach programme run by ESNEFT - a bounded system within which experiences of participants were explored. We were interested in explanatory insights to help us to understand the impacts of the programme. A case study approach using both qualitative and quantitative data sources was therefore identified as the most appropriate approach to achieve these objectives, particularly where the aim was to understand how participants experienced and interpreted the programme [18]. Qualitative interviews offered depth of insight, while survey data provided a broader overview of participant responses. The qualitative interview questions and quantitative survey items were designed to explore the same broad topics. Drawing on both data sources enabled triangulation of findings and a more comprehensive understanding of the outreach intervention. The programme exclusively recruited individuals from under-represented groups; consequently, every participant included in the evaluation met the relevant criteria for under-representation. As noted above, the widening participation criteria are detailed in Appendix 3. Ethical approval for the study was secured from Anglia Ruskin University’s Faculty of Health Medicine and Social Care Research Ethics Panel (Reference number: ETH2425‑7766).

Six semi-structured in-depth interviews were conducted with programme participants during the programme, which sought to elicit deep insights into participants’ experiences of, and attitudes towards, the programme. 'Next AHP' programme participants were given details about the research and a Participant Information Leaflet explaining the study. They were also given the contact email of the research assistant carrying out the interviews and asked to get in touch if they were interested in taking part. Interviews, which were carried out remotely via video conferencing by the second author, took on a conversational style and were loosely steered by a discussion guide (see Appendix 1). Interviews lasted between 17 and 28 min, and on average 24 min. Table 1 gives details of the participants. A relatively small number of interviews is adequate to reach saturation while preserving analytical depth in a study such as this, with a defined focus and relatively homogeneous participants [19].

**Table 1:** Interview participant characteristics

Participant code	Gender	Date of interview	Length of interview	School year
P1	F	18 Sep 25	17 min	Y12
P2	F	21 Nov 25	24 min	Y12
P3	F	21 Nov 25	25 min	Y12
P4	F	26 Nov 25	22 min	Y12
P5	F	2 Dec 25	28 min	Y12
P6	F	12 Dec 25	28 min	Y12

While the qualitative component of the study prioritised depth of understanding, the survey component aimed to generate descriptive insights and to complement the qualitative findings rather than to provide statistical generalisation [20]. Programme participants who had not previously taken part in the in-depth interviews were invited to access an anonymous online survey via a QR code which was made available to them during one of their face-to-face sessions of the 'Next AHP' programme and in a follow-up email circulating the session materials. All participants were year 12 students. The questionnaire (see Appendix 2), which took around five to ten minutes to complete, covered their awareness of AHPs, motivation for joining the programme, experiences of the programme, thoughts on content and timing of the sessions and suggestions for the future. Sixteen surveys were completed.

### Data analysis

In-depth interviews were fully transcribed and analysed inductively using Braun and Clarke’s reflexive thematic analysis approach [21]. Initial in-vivo coding was used to capture participants’ perspectives in their own words and to minimise the imposition of preconceived interpretations [22]. Coding was conducted in NVivo 14, with codes iteratively reviewed and organised into candidate themes. These themes were subsequently refined through discussion by the first three authors to generate the final thematic framework.

Descriptive statistical analyses of the survey data were undertaken in Microsoft Excel, including the calculation of frequencies, percentages, and mean scores, to summarise respondents’ key survey responses.

## Results

### Qualitative insights

In line with the study objectives, participant narratives focused on three key areas: their motivation to join the programme; their experiences of the programme; and the impact that the programme had on them.

### Motivation to join the programme

Participants expressed general and specific motivations for joining the Next AHP programme. General motivations were presented as a broad curiosity about the programme and the benefits it may deliver to them, but also about the NHS more broadly. They consistently pointed to an interest in gaining *a deeper understanding* of the health service and of its constituent parts. They also reflected on what their participation in this programme might say about them to potential employers in the future. Some suggested that demonstrating an interest in healthcare careers by attending the programme could help them to evidence their *dedication and passion*, particularly given that the sessions were delivered at the weekend in their free time:

*I just thought it’d be quite interesting to kind of get to know the different parts of the NHS and like if I wanted to go into that*,* then it was just a good thing to do.* P1.

*I looked at it and I went*,* you know what*,* that that would be very beneficial for me to like to*,* you know*,* have a more deeper understanding*. P6.

*[the programme was] … generally a good experience that you can put on your CV like it shows your dedication and like passion because you’re taking time out of your Saturday to like be a part of this and like get involved which is great.* P4.

Participants also expressed specific curiosity about AHP careers. Often, prior awareness of these professions was low, and a common disclosure was that before the programme, they *didn’t know anything about* AHPs. Some participants articulated a general prior awareness of AHP careers which they wished to develop, for example by understanding *how they worked with other people*. In some cases, participants were surprised to learn that a job they had already been considering was actually an Allied Health Profession:

*I actually didn’t know about AHPs … didn’t know anything about [them] at all.* P3.

*I had heard of them briefly … So I knew kind of what jobs were allied health. But I didn’t understand how they worked with other people and what that kind of entailed* P5.

*So before starting the programme*,* I don’t think I’d ever*,* like*,* heard of Allied health professionals. So when I first came in*,* like for the first session and I started to like realise*… *I actually had thought of a job in AHP*,* even though I didn’t know it was part of AHP… I originally wanted to go into physiotherapy.* P4.

### Experiences of the programme

We identified three sub themes when participants talked about their experiences of the Next AHP programme: that they found it generally enjoyable; that they particularly enjoyed the interactive nature of the programme; and that they were impressed by their interactions with the professionals delivering the sessions through whom they learned a great deal about AHP roles. Overall, it was clear that the participants were strongly and positively impacted by the programme. Starting with general satisfaction, participants enthusiastically expressed all-round enjoyment with the programme, often noting that they found it *informative* and *positive*:

*I loved every single thing about that programme… I found it very*,* very informative because we*,* especially the occupational therapist session and the health and social care session we had*,* I found them very informative and very interesting* P3.

*Overall*,* it’s been a really positive experience. It’s all been good*,* yeah*. P2.

The interactive nature of the sessions, and the opportunity to experience clinical settings, clearly made a significant impression on the students. Experiences of medical settings and technical equipment were recounted with clarity and enthusiasm. Exposure to an operating theatre was *insane* and seeing technical equipment in use was *just so exciting.* This central element of the programme seemed to bring AHP practitioner roles to life for these students.

*So we went into a real-life operating theatre. And then we saw how everything works and I’d never been in an operating theatre before and it was just so exciting* P4.

*I like how it’s interactive and we can see the equipment used. I really liked that part about it* P2.

*… going into a theatre was insane*,* really. You only see stuff like that on TV … It was such a great experience* P5.

Employing practising AHPs as tutors seems to have been key to the interactive experience which students found so enjoyable. Whereas participants anticipated *listening to people talking*, they found much more opportunity to interact with the professionals.

*They’ve made it fun. They haven’t*,* like*,* let you sit there and they just talk at you. They’ve made it really interactive*,* which is really*,* really nice*. P1.

*I thought we’d just be listening to people talking*,* but it’s not been like that and I like how we can*,* like*,* talk to the people and interact.* P2.

Participants particularly commented on tutors’ enthusiasm, especially their willingness to engage fully with the students. Tutors are described as being *really open to* questions and *really passionate* about being part of the programme. Participants emphasised the depth of knowledge they received about AHP roles, often noting that they felt they received a realistic portrayal of the job - *what the profession actually entails*,* good and bad.* Ultimately they described an authentic and motivating experience.

*So I’ve really enjoyed like the sessions where people like explain to me like what they do and how they do and like*,* just also the ability to ask questions*,* they’re really open to questions* P4.

*They’ve all been really passionate. And you can tell they genuinely want to be there and they’re not just doing it because they have to.* P5.

*I think I’ve enjoyed how in depth you kind of go within each profession. It’s not just like surface level ‘we do this’. It’s like you get to see and hear what they’ve what the profession actually entails*,* good and bad …* P1.

Participants described the tutors’ presentations of the realities of working with specific conditions as *pragmatic* and *honest*, highlighting their openness and realism. This approach clearly fostered empathy and understanding in the students as they reflected on, for example, the complexities associated with non-verbal patients and on the skills and adaptability required to deliver person-centred care in the absence of speech.

*It was MND which was quite it was quite emotional*,* I think quite a few people found it very sensitive*,* but she was really pragmatic about it*,* very honest. And it was really interesting to learn about*. P5.

*I came to the realisation that it’s actually quite hard to communicate with a patient who can’t speak. … who’s non-verbal*. P3.

### Impacts of the programme

Participants recognised that the programme fostered belonging and peer connection, by bringing together *like-minded* people who may have had similar ambitions to themselves. They were also positive about the practical and financial support that the programme offered them, such as assistance with travel to the programme sessions and support with applications and interviews. This clearly assisted participants who may otherwise have struggled due to financial or practical barriers.

*It’s like you find like-minded people who all have the same like goals of getting into medicine as you* P4.

*So we spoke about interview techniques … I really appreciate it. I still have the sheet actually up on my wall about how to do it … [and] I got given travel there which was really useful*,* really helpful*,* especially since it was very far from where I live* P5.

According to their accounts, the Next AHP programme positively influenced the career aspirations of multiple participants, who attributed this to their new-found awareness of, and information about, the breadth of AHP opportunities. Several asserted that the programme had made them consider an AHP career path, often encouraged by exposure to practising AHPs as role models and the idea that *I wanted to do that myself.*

*I kind of discovered that the like AHP just spans so much wider than I thought it would do.* P1.

*It’s has me really considering … having a career in*,* AHP too*,* just like seeing where the future takes me … I’m curious about the different aspects of AHP that I could take.* P4.

*I found … I wanted to do that myself* P3.

Reflecting on the programme, they felt that it was helpful for people like themselves who had a broad interest in healthcare careers, but who needed more detail on the opportunities available to them. They also noted the importance of reaching students at the right time, i.e., when they are considering their future plans.

*I think it’s more targeted at people who’d like to go into healthcare*,* but don’t really know what area they want to go into*. P1.

*I’d say it’s suitable still for*,* like anyone who’s either exploring their options*,* they’re not really too sure what they want to do*,* and anyone who might be interested in having a role in the medical field.* P2.

*So I think year twelves people that will be applying to uni*,* or you know*,* going onto their career in about a year’s time*,* college students*,* things like that*. P5.

Participants were asked about their thoughts on ways in which the programme could be improved. While several expressed the view that no improvement was needed, some ideas were raised, including making the sessions longer, to avoid rushing the content, and offering more in-depth assistance on applying for AHP courses.

*I don’t think they could have done anything differently other than make it longer*,* because I think two things*,* two hours or maybe three hours. I don’t think that was long enough* P1.

*I think more help about like how to actually apply to Unis and what they’re expecting from you. I think that would be really helpful for lots of people* P2.

### Quantitative insights

Quantitative data were collected via an online survey to triangulate the qualitative findings and to characterise key respondent responses. We present the quantitative findings using the three broad themes identified in the qualitative findings – motivation, experiences and impact.

### Motivation to join the programme

**Fig. 1:**
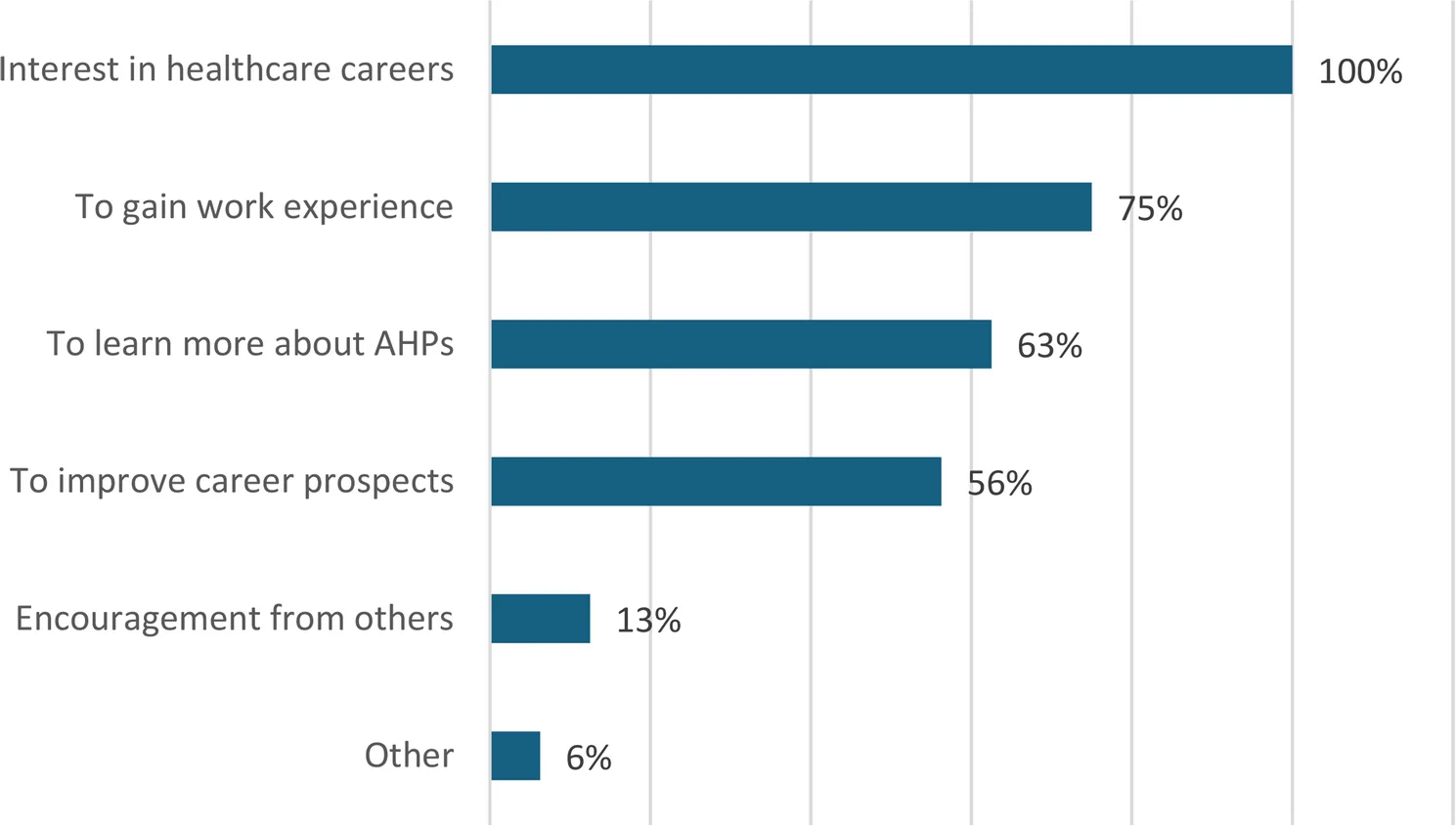
Motivation to join the Next AHP programme Base: 16 programme participants

**Fig. 2:**
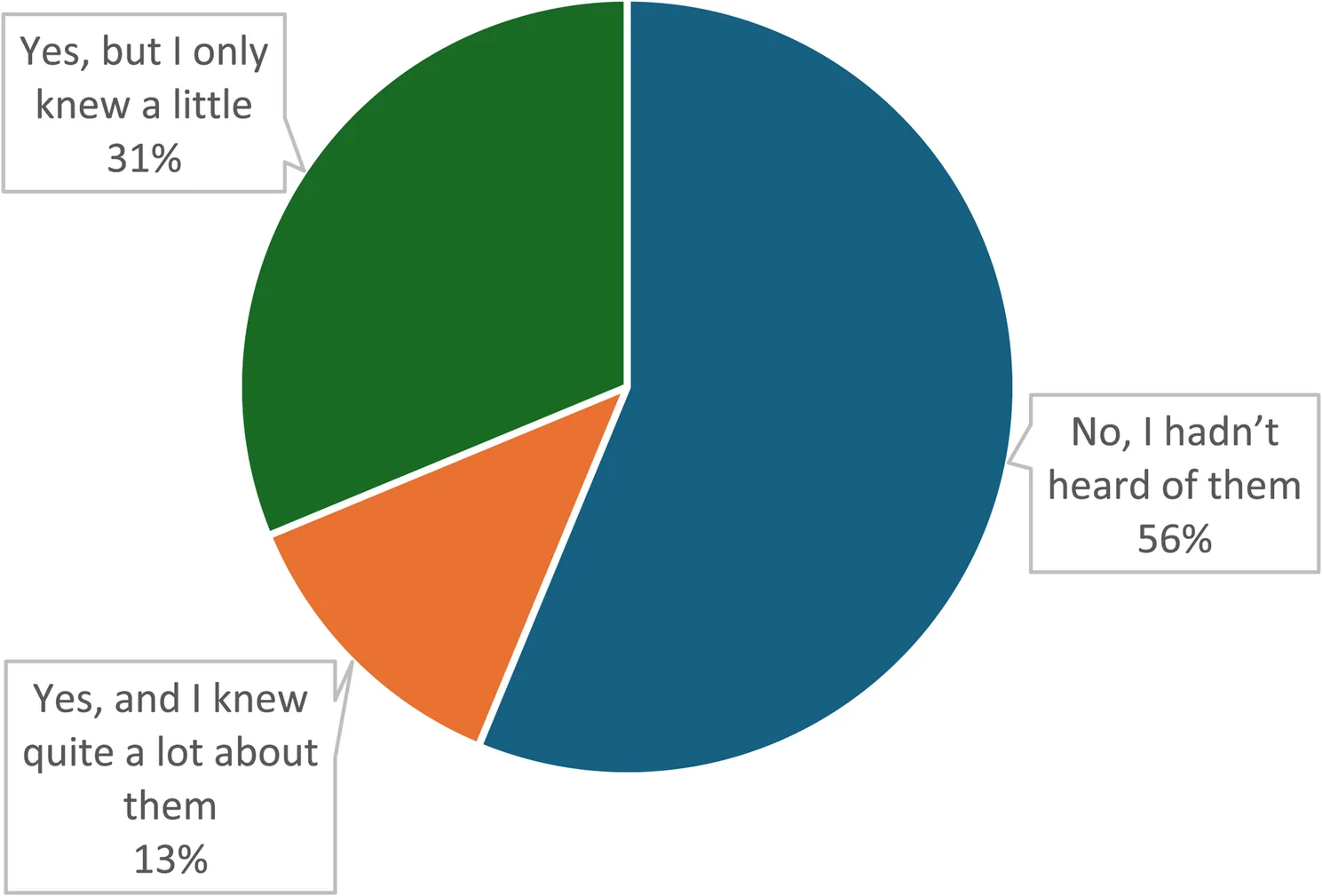
Proportion of participants that had heard of AHPs before the programme Base: 16 programme participants

When asked why they joined the programme all survey respondents said that an interest in healthcare careers motivated their interest. Other motivations included an interest in work experience, learning more about AHPs and improving their career prospects (Fig. 1). As shown in Fig. 2, more than half of the survey respondents said that they had never heard of AHPs before the programme, with a further third saying that they had heard of them but only knew a little about them. Only 13% said they knew a lot about AHPs before joining the programme.

### Experiences of the programme

**Fig. 3:**
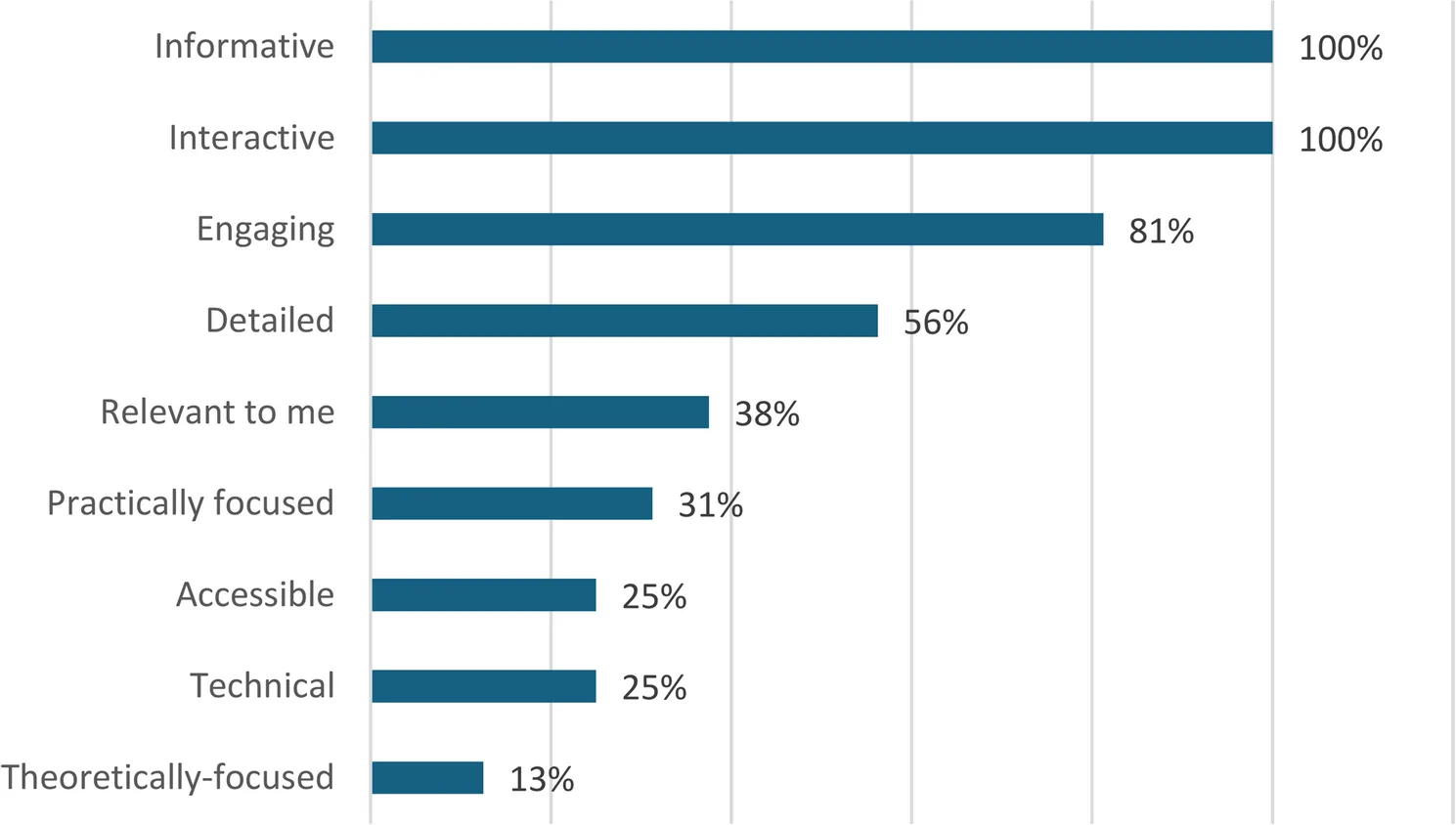
How participants would describe the content of the sessions Base: 16 programme participants

**Fig. 4:**
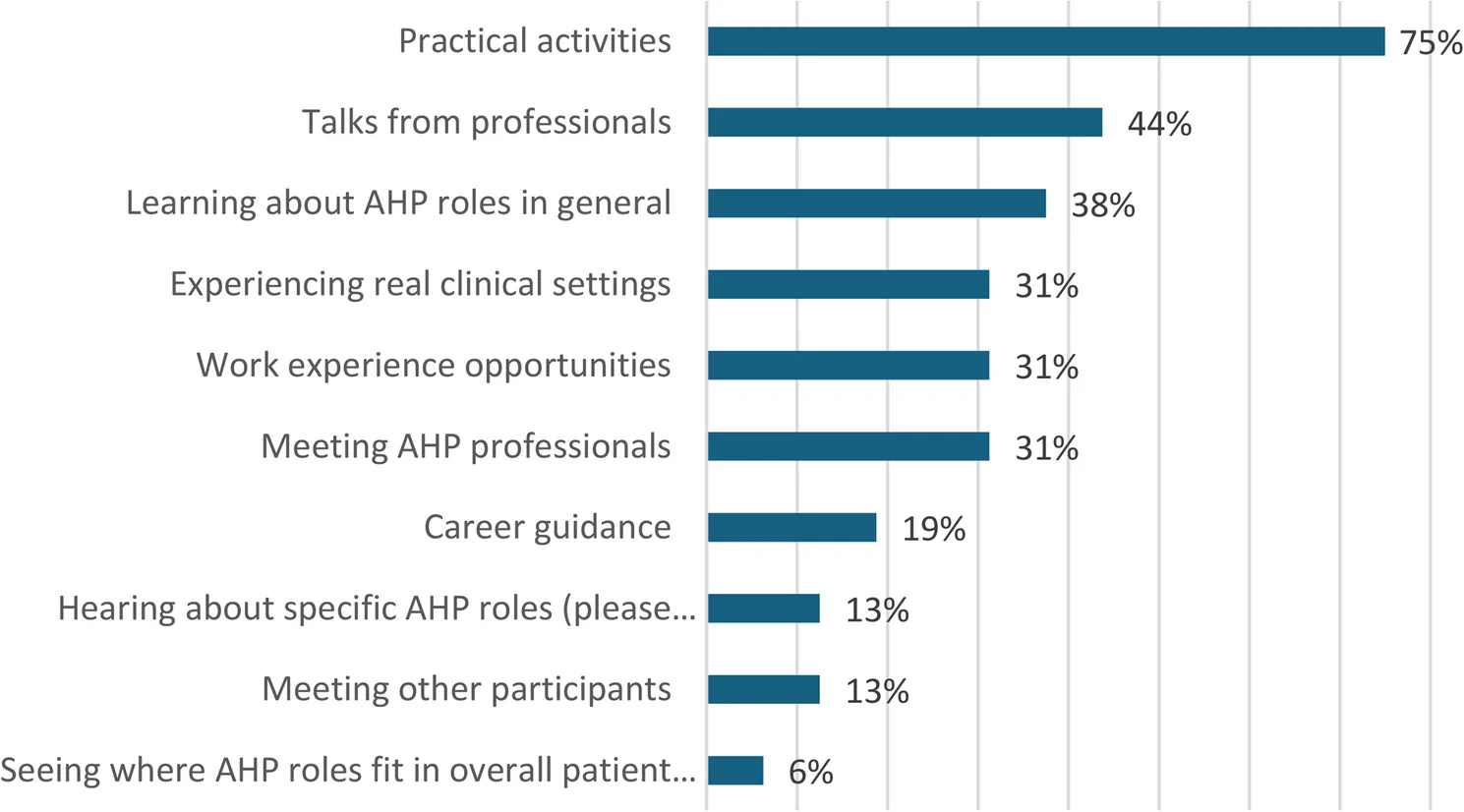
Aspects of the programme participants enjoyed the most Base: 16 programme participants

Focusing on the programme itself, as shown in Fig. 3 the survey data provides strong support for the qualitative finding that participants found the contents of the sessions above all informative and interactive. Most also said it was engaging and more than half pointed to the detailed content. Similarly, when asked which aspects of the programme they enjoyed the most, practical activities was the most popular answer chosen by 75%, also in line with the qualitative data. This was followed by talks from AHP professionals (44%) and learning about AHP roles (38%). Other factors that scored well included, experiencing clinical settings, work experience opportunities and meeting AHPs which were all chosen by 31% of respondents (Fig. 4).

**Fig. 5:**
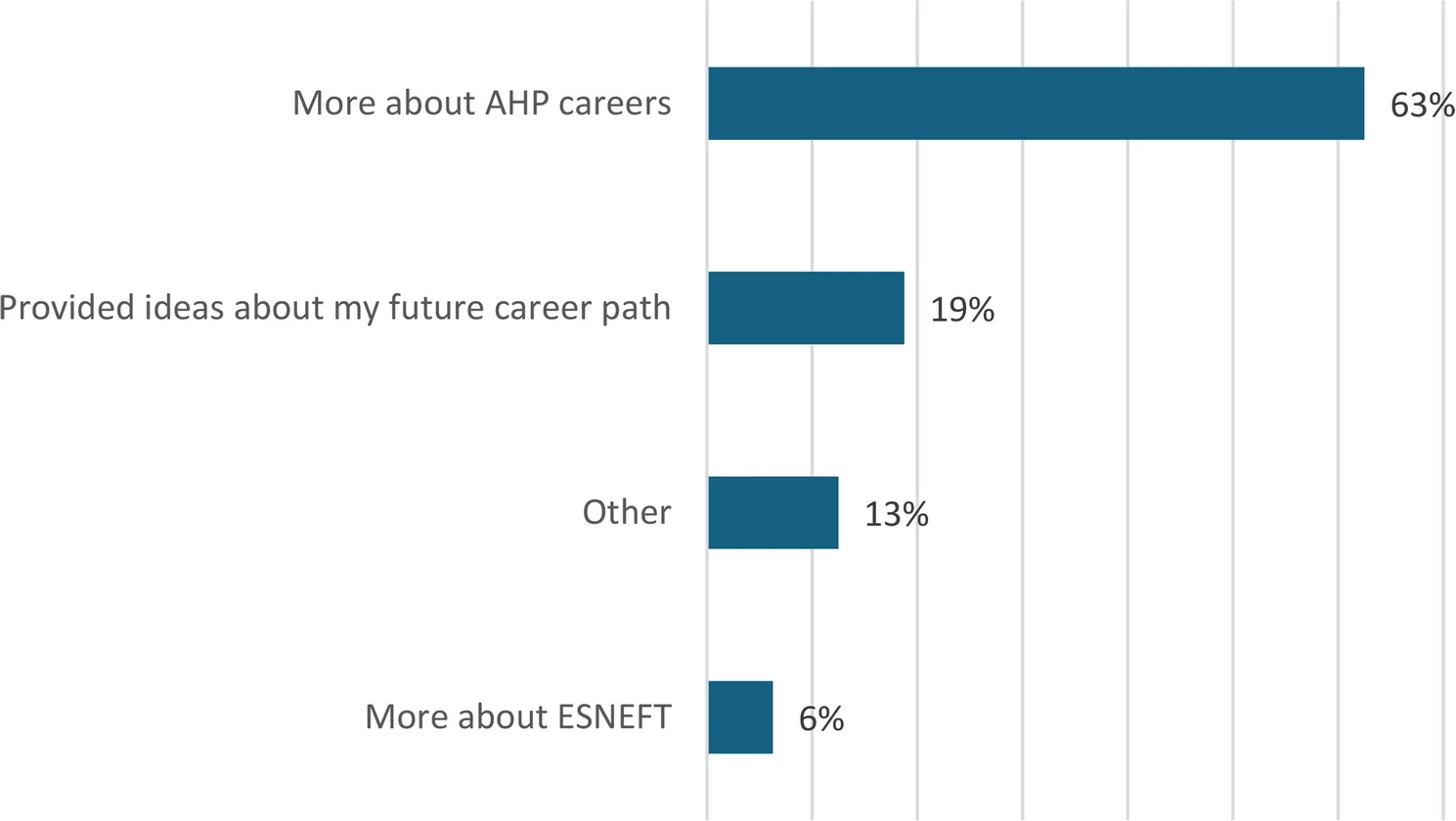
What participants learned during the programme Base: 16 programme participants

Figure 5 indicates that more than 60% of respondents felt they learned more about AHP roles during the programme.

### Impacts of the programme

**Fig. 6:**
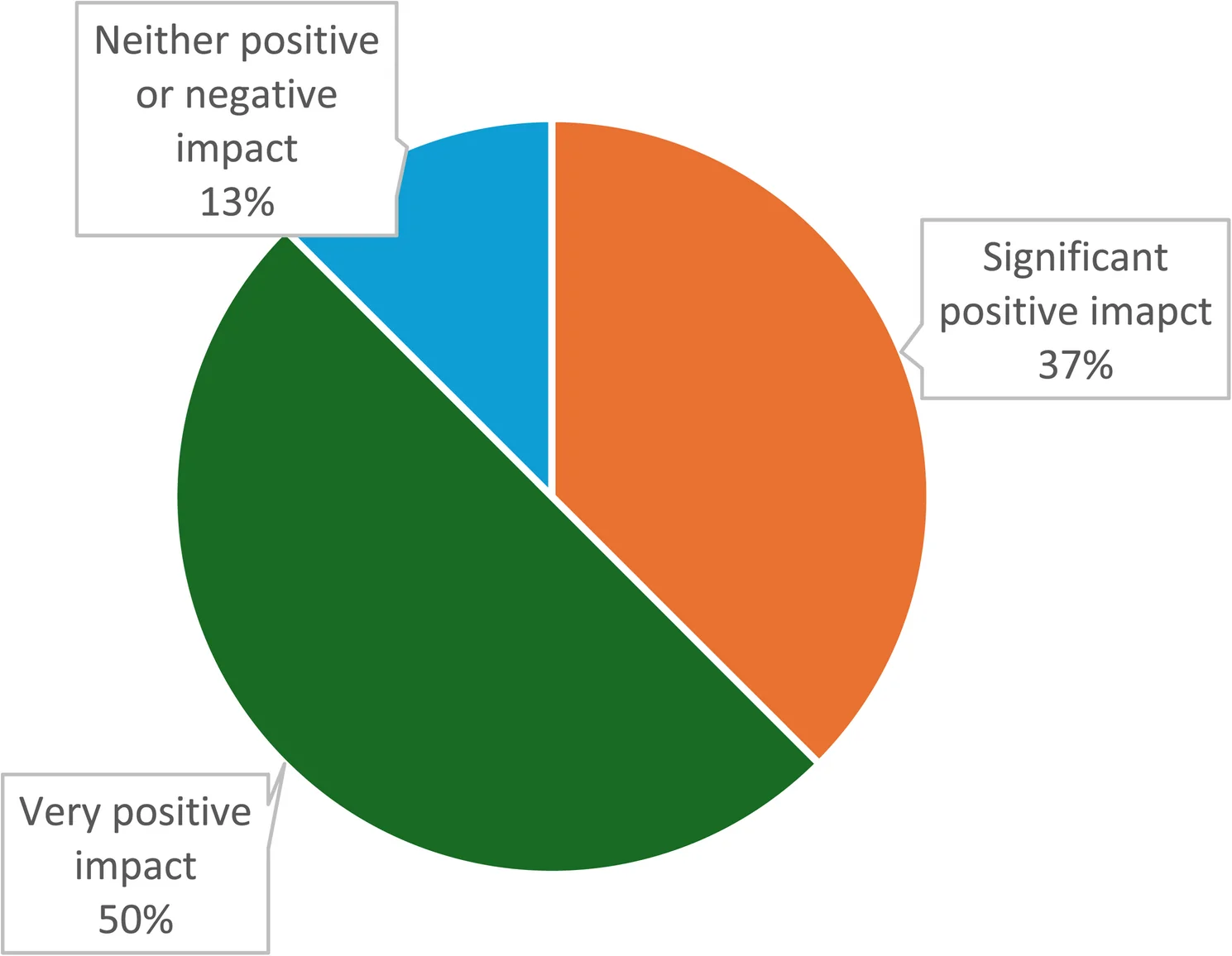
How the programme impacted participants’ interest in pursuing an AHP career Base: 16 programme participants

Asked how the programme impacted on their interest in pursuing an AHP career, half of respondents said that the programme had had a very positive impact, with a further 37% saying it had had a significant positive impact (Fig. 6). Only 13% gave a ‘neither positive nor negative’ response, indicating that the programme had a material effect on the career aspirations of the majority.

## Discussion

This study set out to evaluate the 'Next AHP' intervention. It aimed to understand what motivated students to sign up, to shed light on how they experienced the sessions and to establish what the impact was on their career intentions. It found that students expressed motivations related to general interest in healthcare professions as well as some specific interest in AHP career pathways. But it is clear that a substantial proportion of participants had little or no prior knowledge of AHPs before joining the programme. Given that a central aim of the 'Next AHP' programme was to widen participation by exposing groups who are traditionally not well represented in Allied Health Professions, this lack of awareness may reflect a lack of engagement with AHPs in underrepresented groups. It may also reflect a broader issue with the profile of AHPs in society. Either way, addressing this evident need to raise awareness of AHPs may be a good first step in improving recruitment of AHPs more generally.

A strength of 'Next AHP', and one that participants particularly appreciated, was the interactive nature of the programme and the exposure to clinical settings and equipment. It is also clear that they were very engaged with, and impressed by, the AHPs who delivered the content, particularly with their passion and professionalism. They enjoyed taking part with fellow participants with whom they seemed to feel a connection. Our data suggests that, while a few small adjustments may improve the programme somewhat (longer sessions, more emphasis on university applications) overall it was extremely well received and seemed to provoke a real interest in pursuing AHP careers among those who attended. While we captured career intentions, further longitudinal research might usefully track participants to establish whether these intentions actually translate into actions, in order to establish whether this intervention has a measurable impact on AHP recruitment. The difficulties that the programme had with declining attendance at the start of year 13 seems to have been linked to the need for participants to attend university opens days, and this suggests that beginning the programme earlier (e.g., in year 11 instead of year 12) may be appropriate. This may also allow more time for students to reflect on their university application choices.

Our data suggests that exposing students to clinical environments and giving them direct interactions with AHPs delivered powerful learning experiences, demystifying AHP careers and even enabling students to visualise themselves in these roles. This characterisation is particularly supported by participant narratives that spoke of not previously realising what AHP careers entailed, and yet of being able, after the programme, to imagine themselves working in these roles. It is also evident in the survey data showing that respondents especially enjoyed practical activities, talks from professionals and learning about AHP roles. This direct experience with current professionals seems to have been decisive in participants’ enjoyment of the programme. AHP tutors were presented as approachable role models who were pragmatic and honest and who offered an authentic presentation of their roles. This clearly appealed to many participants, prompting them to reflect on their own future career aspirations and to imagine a future in which they may become an AHP.

Possible Selves Theory [23], developed by Markus and Nurius in 1986, posits that future-oriented self-concepts, or desired future selves, can be highly motivating for individuals because they offer incentives for future behaviour. Possible selves are personalised visions of who an individual hopes, expects, or fears they may become in the future [23]. This thesis offers a valuable framework for understanding how learning interventions such as the 'Next AHP' programme may influence career development. Possible Selves theory proposes that motivation can increase when people can distinctly imagine themselves in future roles. On this account, possible selves are conceptualised as mental links between the present and the future. Individuals strive to attain these future selves through goal setting which in turn influences decisions and behaviour, with the ultimate aim of achieving their desired identities. Markus and Nurius argue that the possible future selves that individuals may imagine for themselves is constrained by a range of factors including their socio-demographic backgrounds, their circumstances, the broader context they inhabit and media depictions. This means that the range of future selves available to individuals, while theoretically infinite, is in practice restricted to ‘the categories made salient by the individual’s particular sociocultural and historical context and from the models, images, and symbols provided by the media and by the individual’s immediate social experiences’ (p954). Possible selves are thus arguably socially determined and may be influenced by an individual’s background and environment. Under-represented groups may, for example, be less likely to see certain professions as achievable for people like themselves, due in part to a lack of role models and limited representation, leading to perceived barriers. Thus, this theory also offers insight into why some groups are underrepresented in Allied Health Professions, while offering the prospect that interventions that boost individuals’ ability to see themselves in a future role may increase their motivation to pursue such a career.

Drawing on this theory, we argue that the programme as a whole, and direct interaction with the AHP tutors delivering the programme in particular, impacted on these participants, and may have allowed them to envisage how such careers might fit with their own identities, enhancing motivation and interest and informing the development of possible future professional selves. This chimes with prior research that points to the way that possible selves can help to raise career aspirations, particularly in underrepresented groups [24].

While the theory has informed research exploring professional identity formation, career aspirations, and educational persistence among healthcare students [25] most studies address healthcare students’ development of future professional identities rather than career choice per se. There is precedent for the use of this framework to investigate interventions which help learners articulate and develop positive professional possible selves in an AHP context. For example, Murphy (2025) found the notion of future selves to be an important motivator for persistence and achievement in healthcare education [26]. However, overall the application of Possible Selves Theory within AHP education is limited.

Our findings suggest that educational interventions which help learners articulate and develop positive professional possible selves may enhance resilience, belonging, and progression among potential AHP students in general, and in under-represented groups particular.

### Limitations

As with all research, this study has limitations. The case study design permitted a detailed exploration of the intervention within its real-world context, eliciting rich insights into participant experiences, implementation processes, and contextual influences on outcomes. However, given the unique and specific context of the case, the findings may have limited transferability beyond the study setting. The small sample sizes for both qualitative and quantitative elements of the study limit generalisability. Furthermore, as an intervention evaluation, the study did not include a control or comparison group, limiting the ability to attribute observed outcomes solely to the intervention. That said, the study was designed to generate in‑depth insights into participants’ experiences rather than to produce broadly generalisable outcomes. The findings should be interpreted as reflecting the experiences and perspectives of the participants involved in the study rather than as representative of the wider population. The consistency of responses across participants suggests the intervention may have meaningful potential, warranting further investigation with larger samples. Findings therefore reflect perceived impact rather than causal effect. Additionally, the evaluation relied on self‑reported perceptions of interest and impact, which may be subject to recall bias or social desirability bias. Future research with larger and more diverse cohorts would be valuable in strengthening the evidence base. Finally, the scheduling of sessions on Saturday mornings may have restricted participation among some eligible individuals with work or caregiving responsibilities. Exploring reasons for non-participation could help inform the design of more accessible interventions.

## Conclusions

We conclude that by offering authentic exposure to clinical environments and practitioners, the 'Next AHP' programme supports positive engagement with AHP careers through multiple interconnected and mutually reinforcing mechanisms. We point in particular to the formation of possible future selves resulting from participation in the programme, through which individuals may come to aspire to career roles they had previously not considered. This study thus contributes to understanding around how early experiential exposure and role models may shape career motivations in allied health professions. While further research is needed, the positive response to the experiential learning offered in the 'Next AHP' programme indicates that ‘grow your own’ approaches to AHP recruitment may benefit from incorporating similar early engagement strategies. Specifically, our study implies that policymakers should consider investing in widening participation initiatives that support under-represented groups to develop awareness of and aspirations towards AHP professions. Embedding career-focused learning interventions more broadly in education programmes may facilitate ongoing professional identity development and career planning. Stronger collaboration between employers, educational institutions, and professional bodies is required to provide accessible pathways, mentoring, and experiential learning opportunities that support recruitment into the AHP workforce.

## Supplementary Information

Supplementary Material 1.

## Data Availability

The datasets generated and analysed during this study are not publicly available to protect participant confidentiality and minimise the risk of identification but are available from the corresponding author on reasonable request.

## Declarations

### Declaration of Helsinki statement

This study was conducted in accordance with the ethical principles of the Declaration of Helsinki. Ethical approval was obtained from the relevant research ethics committee, and all participants provided informed consent prior to participation.
