# Supplementary material for: Promoting allied health profession careers to school students: a case study evaluation of a widening participation outreach intervention
Source: BMC Med Educ. 2026 Aug 7;26:1783. doi: 10.1186/s12909-026-10091-3 (PMC13733008; doi:10.1186/s12909-026-10091-3)
Supplement: Supplementary file 1 [file 12909_2026_10091_MOESM1_ESM.docx]

**Appendix 1: Discussion Guide**

**Objectives:**

1. To evaluate the relevance, coherence, effectiveness and impact of the Next AHP programme, through an exploration of participants’ experiences.
2. To elicit participant’s views on the strengths and limitations of the programme.
3. To understand whether/how Next AHP has influenced participants’ attitudes towards AHP or broader health and care related careers.

**Welcome**: Interviewer to introduce themselves.

**Housekeeping**: Reiterate confidentiality/anonymity assurance as outlined in participant information leaflet.

Tape recording explanation & start recording.

**Introduce purpose of this research:**

- Thanks for taking part in this research, and for confirming that you have read and understood the participant information leaflet and that you consent to the interview being recorded.
- This interview focuses on the Next AHP programme that you are currently enrolled on.
- This research is being carried out by Anglia Ruskin University in partnership with East Suffolk and North Essex NHS Foundation Trust
- We aim to explore your experience of the Next AHP programme.
- I am interested in your thoughts and experiences and ideas. I am not looking for particular ‘right’ or ‘wrong’ answers, but rather to hear your accounts of your experiences and thoughts in your own words.

If there are any questions that you don’t want to answer, please just let me know and we can just move on. Tape recording explanation & start recording.

**Introduction/warm up:**

- Had you heard of Allied Health Professionals before you got involved with this programme? What did you know about them?
- Had you thought at all about a career as an Allied Health Professional before joining this programme?
- Can you tell me a bit about how you came to be involved in this programme?
- When and how did you hear about it?
- Why did you decide to apply?
- How many sessions have you attended?
- Do you plan to finish the programme? If not, is there any particular reason?

**General feelings about the programme:**

- Can you tell me a bit about the different sessions you have attended so far? How have you found them in general?
- Have they been as you expected? If not, why? What were you expecting?
- What sorts of things have you covered?
- How have the sessions worked?
- What have you learned? (About AHPs? About ESNEFT (the trust)? About yourself?)

**More in-depth feelings about the programme:**

- What have you particularly enjoyed about the programme? Why?
- Is there anything that you have not enjoyed? Why?
- Has any session been especially memorable? Or especially useful? Why?
- Was any session particularly good? Or particularly bad, in your view? Why? How could it have been improved?
- How do you feel about the people who have delivered the sessions? Did you find them interesting?
- Is there anything you have not covered in the programme so far that you would have liked to cover?
- How do you feel about the people who are doing the programme with you? Would you say they are similar to you or different from you? Do you feel you have anything in common with them? Have you made new friends?
- Who do you think this programme is targeted at?
- Who do you think it is suitable for?
- Has this programme made you think about pursuing a career as an AHP or not? Why/ why not?
- Have you done any (AHP/healthcare related) work experience? What was it like? How did you find it? What was the best bit? What was the worst bit? Has it made you think differently about AHP careers?

**Close:**

- Before we finish, is there anything else that you would like to say about the programme? Has our discussion brought anything else to mind?
- Thank you for taking part in this project. If anything else occurs to you, please do get in touch. GIVE CONTACT DETAILS/POINT TO THE PARTICIPANT INFO FORM
- Would you be interested in taking part in future research related to this programme? CAPTURE DETAILS IF YES
- The study will be continuing for some time, in a number of phases. Would you like a copy of the research report when it becomes available? CAPTURE DETAILS IF YES

**Appendix 2: Questionnaire**

**Allied Health Professionals Programme – Participant Survey**

Thank you for taking the time to complete this short survey. Your feedback is very valuable and will help shape the future of this programme. The survey should take around **5 minutes** to complete.
All responses will be kept confidential.

**Section 1: Awareness and Motivation**

1. **Before taking part in this programme, had you heard of Allied Health Professionals (AHPs)?**

- ☐ Yes, and I knew quite a lot about them
- ☐ Yes, but I only knew a little
- ☐ No, I hadn’t heard of them

2. **Before joining this programme, had you ever thought about a career as an AHP?**

- ☐ Yes, I had a clear interest
- ☐ I’d considered it but didn’t know much
- ☐ Not really
- ☐ No, not at all

3. **How did you first hear about this programme?**

- ☐ School or college
- ☐ Friends or family
- ☐ Social media
- ☐ AHP event or talk
- ☐ Careers adviser or teacher
- ☐ Other (please specify): ____________

4. **What made you decide to apply for the programme?** *(Select all that apply)*

- ☐ Interest in healthcare careers
- ☐ Encouragement from others
- ☐ To gain work experience
- ☐ To learn more about AHPs
- ☐ To improve career prospects
- ☐ Other (please specify): ____________

5. **Do you plan to complete the full programme?**

- ☐ Yes
- ☐ No
- ☐ Not sure yet

6. *(If No or Not sure)* **Please tell us briefly why:**

- [Free text box]

**Section 2: Your Experience of the Programme**

7. **Overall, how would you rate the session you’ve attended so far?**

1= Poor – 5= Excellent

8. **Was the session what you expected?**

- ☐ Yes, completely
- ☐ Partly
- ☐ Not really
- ☐ Not at all

9. **How would you describe the content of the session?** *(Select all that apply)*

- ☐ Informative
- ☐ Interactive
- ☐ Engaging
- ☐ Repetitive
- ☐ Not relevant to me
- ☐ Relevant to me
- ☐ Technical
- ☐ Practically focused
- ☐ Detailed
- ☐ Theoretically-focused
- ☐ Accessible
- ☐ Other (please specify): ____________

10. **How would you describe the people delivering the sessions?**
    1. = Not very engaging – 5= Very engaging

**Section 3: Highlights and Suggestions**

11. **Which aspects of the programme have you enjoyed the most? (select 3 options)**

- ☐ Practical activities
- ☐ Talks from professionals
- ☐ Meeting other participants
- ☐ Learning about AHP roles in general
- ☐ Work experience opportunities
- ☐ Experiencing real clinical settings
- ☐ Career guidance
- ☐ Meeting AHP professionals
- ☐ Seeing where AHP roles fit in overall patient care
- ☐ Hearing about specific AHP roles (please specific which role) _______________
- ☐ Other (please specify): ____________

12. **Is there anything you have not enjoyed?**

- ☐ Yes
- ☐ No
- ☐ Not sure

13. *(If Yes)* **Please tell us briefly what and why:**

- [Free text box]

**Section 4: Scheduling and Accessibility**

14. **Which days of the week would you find most convenient for programme sessions? *(Select up to two)***

- ☐ Monday
- ☐ Tuesday
- ☐ Wednesday
- ☐ Thursday
- ☐ Friday
- ☐ Saturday
- ☐ Sunday

15. **What time of day would you generally prefer sessions to take place?**

- ☐ Morning (before 12 pm)
- ☐ Early afternoon (12–3 pm)
- ☐ Late afternoon (3–6 pm)
- ☐ Evening (after 6 pm)

16. **How long would you prefer each session to last?**

- ☐ Full day (around 6–7 hours)
- ☐ Half day (around 3–4 hours)
- ☐ Short session (1–2 hours)
- ☐ It depends on the topic

17. **How often would you ideally like sessions to run?**

- ☐ Fortnightly (every two weeks)
- ☐ Monthly
- ☐ Other (please specify): ____________

18. **Is there anything that would make it easier for you to attend sessions (e.g. travel, timing, format)?**

- [Free text box]

**Section 5: Impact and Next Steps**

19. **Who do you think this programme is most suitable for?**

- ☐ People interested in healthcare
- ☐ Anyone exploring career options
- ☐ People already set on an AHP career
- ☐ People interested in AHP careers
- ☐ I’m not sure

20. **Have you done any AHP or healthcare-related work experience outside this programme?**

- ☐ Yes
- ☐ No
- ☐ I don’t know

21. *(If Yes)* **How would you describe that experience overall?**

1 = Poor – 5= Excellent

**Section 5: Final Comments**

22. **Is there anything else you would like to share about your experience of the programme?**

- [Free text box]

23. **Would you be happy to be contacted for future research about this programme?**

- ☐ Yes (please provide contact details): ____________
- ☐ No

24. **Would you like to receive a copy of the research report once it is available?**

- ☐ Yes (please provide contact details): ____________
- ☐ No

**Appendix 3: Widening Participation Criteria**

• Attended a school for GCSEs which has an attainment 8 score (in England), or a

Capped 9 points score (in Wales) below the respective national average.

OR

• You have experienced local authority care for a period of longer than 3 months

within the last 5 years*

OR

Attended a non-selective state school for GCSEs and A-levels and meet one of the

following criteria

• Live in an area with an Index of Multiple Deprivation (IMD) in the bottom 20%

• In receipt of free school meals, Pupil Premium funding and/or discretionary

school/college payments, which are directly linked to financial hardship.

• You have a household income <£42,000 per year.

• You or your parent(s)/guardian(s) are in receipt of means tested benefits**

• You are a person who has sole unpaid caring responsibility for another person.

• You are in receipt of a UCAT bursary.

• You have refugee status.

• You are a young person who is estranged from your family.

• You are a member of the gypsy or travelling community

* A Care Leaver is someone who has been in the care of the Local Authority for a

period of 13 weeks or more spanning their 16th birthday. A person is Care Experienced

if they have been or are currently in care or from a looked after background at any

stage of their life, including adopted children who were previously looked after. Care

may have been provided in a number of ways or settings such as foster care, kinship

care, residential care or through being looked after at home with a supervision

requirement.

**Parental benefits will not be considered for applicants over the age of 21
